# Natural killer cell infusion for cytomegalovirus infection in pediatric patients with Wiskott-Aldrich syndrome following cord blood transplantation: A case report and literature review

**DOI:** 10.3389/fmed.2022.988847

**Published:** 2022-10-10

**Authors:** Yongwei Yue, Lijun Meng, Jing Ling, Liyan Fan, Yanlei Zhang, Yixin Hu, Alex H. Chang, Shaoyan Hu

**Affiliations:** ^1^Department of Hematology & Oncology, Children's Hospital of Soochow University, Suzhou, China; ^2^Clinical Translational Research Center, Shanghai Pulmonary Hospital, Tongji University School of Medicine, Shanghai, China; ^3^Shanghai YaKe Biotechnology Ltd., Shanghai, China

**Keywords:** NK cell infusion, cytomegalovirus infection, Wiskott-Aldrich syndrome, cord blood transplantation, case report

## Abstract

NK cells have important functions in resisting cytomegalovirus infection, as they proliferate after viral infection and have certain immunological memory. Here, we report infusion of haploid donor-derived natural killer cells to treat two pediatric patients with Wiskott-Aldrich syndrome (WAS) who were infected with cytomegalovirus after cord blood transplantation (CBT), which successfully cleared the viral infection in both patients.

## Introduction

Natural killer cells (NK cells) are an essential component of innate immunity and play important antiviral and antitumor roles in addition to maintaining immune homeostasis. They also have important functions in resisting cytomegalovirus infection, as they proliferate after viral infection and have certain immunological memory. Here, we report infusion of haploid donor-derived natural killer cells to treat two pediatric patients with Wiskott-Aldrich syndrome (WAS) who were infected with cytomegalovirus after cord blood transplantation (CBT), which successfully cleared the viral infection in both patients.

## Case 1

**Figure 1:**
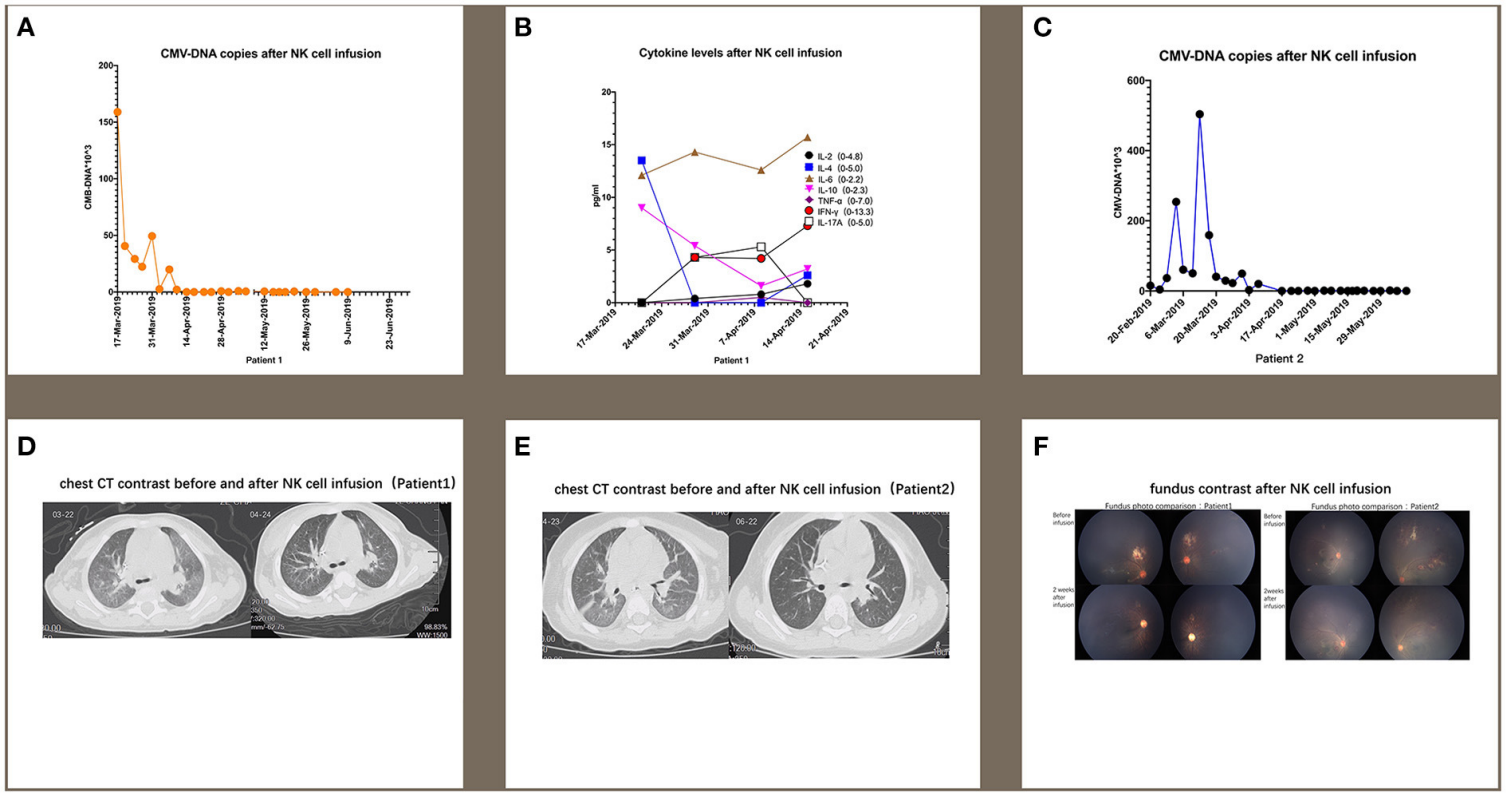
For patient 1, the number of CMV DNA copies **(A)** changes in cytokine levels **(B)**, and chest computed tomography (CT) obtained before and after cell infusion **(D)** are presented in this figure. For patient 2, the changes in CMV DNA copy number **(C)** and chest CT **(E)** after NK cell infusion are also presented. **(F)** indicates that CMV infection-related retinitis improved after NK cell infusion in both patients. NK cells were expanded directly from peripheral blood mononuclear cells (PBMCs) collected from two haploidentical donors, and cultured for 3 weeks by adding irradiated artificial antigen presenting cells (aAPC-NKElixir^®^, a proprietary NK cell expansion technology from Shanghai YaKe Biotechnology Ltd.) weekly. Fresh NK cells were infused on day 21. The remaining NK cells were cryopreserved until infusion. The cryopreserved NK cells were prepared for infusion by thawing in a 37°C water bath, washing once with infusion buffer (0.5% human serum albumin in Plasmalyte A) and resuspending the final cell dose in 100 mL of infusion buffer.

The first patient was a male who was diagnosed as WAS because of low platelet since newborn. The blood test showed a white blood cell count of 9.82 × 10^9^/L, neutrophil 2.23 × 10^9^/L, hemoglobin 109 g/L, and platelet 62 × 10^9^/L. Bone marrow aspiration (BMA) revealed a neutrophil maturation, high eosinophil count at 10%, active erythroid and lymphocyte proliferation with immature lymphocytes accounting for 2.5% of marrow nucleated cells, reduced platelet distribution, and active megakaryocyte proliferation showing mild maturity disorder. Genetic analysis revealed a hemizygous pathogenic variant in the *WAS* gene on chr X-48544528 (c.559 + 5G>A). Before receiving cord blood transplantation (CBT), the patient had been treated with intravenous immunoglobulin (IVIG), and with antibiotics and platelet infusion when needed. When the patient was older than 6 months, he showed a slow growth and worse platelet infusion. When he grew at 8 months and the weight kept 8 kg for 2 months, he received CBT. The conditioning regimen was Busulfan (Bu) + Cyclophosphamide (CTX) + Fludarabine (Flu) + anti-thymocyte globulin (ATG), and tacrolimus (FK506) plus mycophenolate mofetil (MMF) were prescribed as prophylaxis of graft-vs.-host-disease (GVHD). The unrelated cord blood was B-type Rh (+), HLA 8/10, with a CD34^+^ cell count of 8.07 × 10^5^/kg, and total nucleated cell count of 10.6 × 10^7^/kg. The granulocytes and platelets were engrafted at 12 and 30 days after cord blood infusion, respectively. This patient developed cytomegalovirus (CMV) infection with pneumonia and retinitis 2 months after receiving CBT, for which he was treated with ganciclovir, foscarnet sodium, and lyophilized IVIG for 7 weeks. However, there was a significant increase in the copy numbers of CMV-DNA when examined by real-time-PCR after treatment compared with that prior to treatment (5.04 × 10^5^ measured on 2019-03-13 vs. 3.08 × 10^3^ measured on 2019-02-03). Therefore, from 2019-03-19 to 2019-04-18 (Table 1), we performed weekly natural killer (NK) cell infusion for six times, using NK cells expanded from the peripheral blood (PB) of the patient's father after the father was confirmed positive for CMV IgG. The weight of the patient was 9 kg when the NK cell therapy began, and a total number of 5.4 × 10^8^ NK cells was infused. The proportion of CD3^−^/CD56^+^ NK cells was 79.71%, whereas that of CD3^+^/CD56^+^ NKT cells was 15.8%. The number of CMV DNA copy numbers (Figure 1A), changes in cytokine levels (Figure 1B), and chest computed tomography (CT) obtained before and after cell infusion (Figure 1D) are presented in Figure 1. The CMV DNA copy numbers in the PB of the patient decreased after treatment. Concomitantly, the chest CT improved after NK cell infusion, and there was no obvious GVHD manifestation. As shown in Figure 1B, except for slightly elevated interleukin (IL)-6 levels, no significant changes in other cytokine levels were observed.

**Table 1:** The detailed information for NK cells infusion.

	**Patient 1**						
	**1**	**2**	**3**	**4**	**5**	**6**	
Date of infusion	3/19/2019	3/21/2019	3/28/2019	4/4/2019	4/11/2019	4/18/2019	
Dosage (Cells/Kg)	2 × 10E6	8 × 10E6	1 × 10E7	1 × 10E7	2 × 10E7	1 × 10E7	Weight: 9 KG
Total number (Cells)	0.18 × 10E8	0.72 × 10E8	0.9 × 10E8	0.9 × 10E8	1.8 × 10E8	0.9 × 10E8	5.4 × 10E8
NK (CD3-/CD56+)	79.71%						
NKT (CD3+/CD56+)	15.80%						
**NK/NKT**	**94.51%**						
T (CD3+/CD56-)	4.53%						
	**Patient 2**						
	**1**	**2**	**3**	**4**	**5**	**6**	
Date of infusion	4/15/2019	4/22/2019	4/28/2019	5/7/2019	5/16/2019	5/22/2019	
Dosage (Cells/Kg)	1 × 10E7	1 × 10E7	1 × 10E7	1 × 10E7	1 × 10E7	1 × 10E7	Weight: 12 KG
Total number (Cells)	1.2 × 10E8	1.2 × 10E8	1.2 × 10E8	1.2 × 10E8	1.2 × 10E8	1.2 × 10E8	7.2 × 10E8
NK (CD3–/CD56+)	62.50%						
NKT (CD3+/CD56+)	17.80%						
**NK/NKT**	**80.30%**						
T (CD3+/CD56–)	19.70%						

The release criteria with respect to screening for infections were negative for bacteria, fungi, mycoplasma, and with endotoxin level below 0.25 EU/ml.

## Case 2

The second patient was also a male and had the same history as the first patient. He had a low platelet (34 × 10^9^/L) after he was born and BMA revealed decreased granulocyte proliferation with predominantly multi-lobulated nuclear granulocytes, the number of late erythrocytes was reduced partly, and the percentage of lymphocytes was 44%. All cells exhibited normal morphology. No megakaryocytes were observed in the film, and platelets were also rarely observed. Genetic testing identified a maternal-derived hemizygous pathogenic variant in *WAS* on chr X-48542673 NM000377; exon 2 (c.134C>T; p.T45M). After being diagnosed with WAS, the patient was administered IVIG and platelet infusion. At 12 months old, the patient was administered CBT with the same conditioning regimen and GVHD prophylaxis as the first patient. The graft cord blood was A-type Rh (+), HLA 7/10, with TNC count of 5.4 × 10^7^/kg and CD34^+^ cell count of 1.80 × 10^5^/kg. The engraftment time for granulocytes and platelets was at 14 and 20 days. One month after transplantation, the patient developed CMV infection coupled with pneumonia and retinitis. There was insufficient therapeutic improvement after combined antiviral therapy using ganciclovir, foscarnet sodium, and lyophilized IVIG for 6 weeks, with a significant increase in CMV DNA copy number compared with that before antiviral treatment (1.99 × 10^4^ measured on 2019-04-07 vs. 4.01 × 10^3^ measured on 2019-02-20). Therefore, we performed weekly infusions of NK cells six times from 2019-04-15 to 2019-05-22, using cells expanded from the PB of the patient's father with CMV IgG positive (Table 1). The patient weighed 12 kg at the time of NK cell infusion, and the total number of NK cells infused was 7.2 × 10^8^. Among the infused cells, the proportion of CD3^−^/CD56^+^ NK cells was 62.5%, CD3^+^/CD56^+^ NKT cells 17.8%, and that of CD3^+^/CD56^−^ T cells 19.7%. The changes in CMV DNA copy number (Figure 1C) and chest CT (Figure 1E) after NK cell infusion are presented in Figure 1. The peripheral blood CMV DNA copy number and chest CT improved after the NK cell infusion, and there was no obvious GVHD.

In addition, supplemented by regular intravitreal injection of ganciclovir, CMV infection-related retinitis improved after NK cell infusion in both patients (Figure 1F).

## Discussion

Here, we reported the use of NK cell infusion in treating two children with WAS who developed CMV infection after CBT. Interestingly, their CMV copy number did not respond to antiviral drugs such as ganciclovir, foscarnet sodium, and immunoglobulin, and they further presented with CMV-related pneumonia and retinitis. Upon receiving haploidentical NK cell infusion, all of their symptoms improved significantly without any obvious adverse effects. Our results indicate that NK cell infusion therapy is an optional regimen for WAS compounded by CMV disease.

Patients with WAS are more likely to develop CMV disease that can last a relatively long time after HSCT, particularly CBT. It is known that the immune system of WAS patients is impaired. Sha et al. used a mouse model to demonstrate that the production of interleukin-2 and granzyme B from CD8^+^ T lymphocytes decreases, whereas autophagy of CD8^+^ T lymphocytes increases after murine cytomegalovirus infection, suggesting that lack of WASP may lead to CD8+ T lymphocyte function defects (1). Lang et al. reported reduced interferon-I production by dendritic cells in *WAS* knockout mice, in addition to impaired CD8^+^ T lymphocyte proliferation. Under the premise that CD8^+^ T lymphocytes are functionally defective, CMV clearance was compromised in this group of mice, and they exhibited aggravated immune damage (2). Patients with WAS, a primary immunodeficiency caused by the lack of or reduction in the levels of the WAS protein, usually take longer to reach immunity restoration after CBT and are prone to repeated infections. As a result, CMV infections are commonly observed after CBT, with patients more likely to develop CMV resistance after CBT, resulting in poor response to conventional antiviral treatment (3, 4).

With the increasing adoption of antiviral preemptive treatment, guided by results from polymerase chain reaction, the incidence of CMV disease in the early stage has dropped from 18 to 5%, and the mortality rate has dropped from 46 to 17% (5, 6). However, CMV disease caused by virus activation can still cause fatal multi-organ damage, including pneumonia, enteritis, and retinitis (7). Among the risk factors for CMV resistance proposed by El Chaer et al., delayed immune reconstitution, CBT, young age, and congenital immunodeficiency syndrome are host factors, whereas viral factors include high viral load and whether CMV load increase occurred simultaneously with treatment and whether it responded to standard treatment (8).

Common drugs for treating CMV infection include ganciclovir, valacyclovir, foscarnet, letermovir, and valganciclovir (9). For patients with CMV disease, the European Conference on Infections in Leukemia (ECIL) guidelines recommend intravenous ganciclovir or foscarnet treatment, with cidofovir as the second or third line of treatment. For patients with CMV-related pneumonia, immunoglobulins or CMV-specific immunoglobulins can also be used. Intravenous ganciclovir or foscarnet is recommended for patients with CMV-related retinitis, whereas CMV-specific T cells are recommended for those with refractory CMV infection (10). Recent studies have shown that letermovir can be used as an alternative drug for refractory CMV infection or for treating CMV resistance (11, 12). However, maribavir, brincidofovir, and the DNA vaccine ASP113 failed to improve CMV-related outcomes in phase III clinical trials (13–15). Leflunomide has been investigated as treatment for multidrug-resistant or refractory CMV infections and is currently recommended as an adjuvant for conventional anti-CMV therapy (8, 16, 17). However, there are many limitations when utilizing these therapeutic options in children.

CMV-seropositive patients can be diagnosed based on an increase in CD94/NKG2C^+^ cell count. Although these cells do not express the inhibitory receptor CD94/NKG2A, they express low levels of the activating receptors NKp30 and NKp46 (18), suggesting that CMV infection may remodel NK surface receptors. Rolle et al. found that upon human CMV infection, the expression of HLA-E in infected cells was upregulated and the secretion of IL-12 by monocytes increased, which further resulted in the expansion of CD94/NKG2C^+^ cells (19). In addition, chronic infection with hepatitis B virus, human immunodeficiency virus, Hantavirus, and chikungunya virus is also accompanied with CD94/NKG2C^+^ NK cell expansion and long-term survival. This suggests that NK cells have a broad-spectrum antiviral effect and may possess certain memory functions (20–22). In most antitumor studies, the average NK cell infusion dose at 1 × 10^7^/kg is both safe and effective (23–26). In our study, we chose the same average dose and demonstrated its anti-CMV activity.

## Conclusion

Although a variety of drugs are available for the prevention and treatment of CMV infection, CMV resistance and disease are still serious threats faced by patients after HSCT, especially for CBT. Current methods to enhance the cytotoxicity of NK cells include *in vitro* cytokine expansion (IL-2, IL-15, and IL-12), helper cell co-culture stimulation, and targeted drug research using inhibitory receptors KIR and NKG2A as targets. With technological improvement and further in-depth elucidation of the cellular mechanism of CMV infection, combined with continuous clinical research, NK cell infusion may be reliably used for the treatment of CMV infection after CBT.

## Data availability statement

The original contributions presented in the study are included in the article/supplementary material, further inquiries can be directed to the corresponding author/s.

## Ethics statement

The studies involving human participants were reviewed and approved by Children's Hospital of Soochow University, 92 Zhongnan Street, Suzhou 215000, Jiangsu, China. Written informed consent to participate in this study was provided by the participants' legal guardian/next of kin.

## Author contributions

All authors listed have made a substantial, direct, and intellectual contribution to the work and approved it for publication.

## Funding

This work was supported by National Natural Science Foundation of China (Nos. 81970163 and 81770193) and Suzhou Key Lab (No.SZS201615) to SH, partially supported by National Key Basic Research Program of China (No. 2016YFC1303403), National Natural Science Foundation of China (No. 81272325) to AC, National Natural Science Foundation of China (No. 81700165) to YH. This work was also supported by Jiangsu key project (Nos. BE2019672 and BE2021654) and National Clinical Research Center for Hematological Disorders (2020ZKPB02).

## Conflict of interest

AC is a founding member of Shanghai YaKe Biotechnology Ltd., a biotechnology company focused on research and development of tumor cellular immunotherapy. The remaining authors declare that the research was conducted in the absence of any commercial or financial relationships that could be construed as a potential conflict of interest.

## Publisher's note

All claims expressed in this article are solely those of the authors and do not necessarily represent those of their affiliated organizations, or those of the publisher, the editors and the reviewers. Any product that may be evaluated in this article, or claim that may be made by its manufacturer, is not guaranteed or endorsed by the publisher.
